# RPAS Forensic Validation Analysis Towards a Technical Investigation Process: A Case Study of Yuneec Typhoon H

**DOI:** 10.3390/s19153246

**Published:** 2019-07-24

**Authors:** Fahad E. Salamh, Umit Karabiyik, Marcus K. Rogers

**Affiliations:** Department of Computer and Information Technology, Purdue University, West Lafayette, IN 47907, USA

**Keywords:** digital forensics, drone forensics, forensic process, Yuneec Typhoon H

## Abstract

The rapid pace of invention in technology and the evolution of network communication has produced a new lifestyle with variety of opportunities and challenges. Remotely Piloted Aerial Systems (RPAS) technology, which includes drones, is one example of a recently invented technology that requires the collection of a solid body of defensible and admissible evidence to help eliminate potential real-world threats posed by their use. With the advent of smartphones, there has been an increase in digital forensic investigation processes developed to assist specialized digital forensic investigators in presenting forensically sound evidence in the courts of law. Therefore, it is necessary to apply digital forensic techniques and procedures to different types of RPASs in order to create a line of defense against new challenges, such as aerial-related incidents, introduced by the use of these technologies. Drone operations by bad actors are rapidly increasing and these actors are constantly developing new approaches. These criminal operations include invasion of privacy, drug smuggling, and terrorist activities. Additionally, drone crashes and incidents raise significant concerns. In this paper, we propose a technical forensic process consisting of ten technical phases for the analysis of RPAS forensic artifacts, which can reduce the complexity of the identification and investigation of drones. Using the proposed technical process, we analyze drone images using the Computer Forensics Reference Datasets (CFReDS) and present results for the Typhoon H aerial vehicle manufactured by Yuneec, Inc. Furthermore, this paper explores the availability and value of digital evidence that would allow a more practical digital investigation to be able to build an evidence-based experience. Therefore, we particularly focus on developing a technical drone investigation process that can be applied to various types of drones.

## 1. Introduction

According to the National Institute of Standards and Technology (NIST) [1], criminal drone operations are rapidly increasing and criminals are constantly developing new approaches. These criminal operations include invasion of privacy, drug smuggling [2], terrorist operations [3], and the disruption of critical infrastructure [3]. An example of this is the recent (December 2018) incident at Gatwick Airport in the United Kingdom involving unauthorized drone operation in the airport flight path and on airport property, impacting thousands of travelers [4]. The U.S. Federal Aviation Administration (FAA) forecasts that the number of Unmanned Aerial Systems (UAS) will reach 7 million in 2020 [5]. The Remotely Piloted Aerial System (e.g., drone), is an example of a widely used technology that requires the collection of a solid body of evidence to help eliminate potential real-world threats generally posed by malicious RPAS operations. It is necessary to apply digital forensic techniques and procedures to be able to generate a unified technical process that aids in the forensic analysis of drones in order to create a line of defense against aerial incidents.

This paper examines digital evidence and artifacts using the benchmark drone forensic images of the Yuneec, Inc. Typhoon H drone, which was recently involved in delivering contraband content to a prison [2]. Flying the Typhoon H drone does not require the use of a smartphone, as it has a built-in “ST16S” ground station controller. This is not the case with every drone, as some drones require use of a smartphone as a controller. However, our research provides a technical process that would fit the majority of drones. This proposed drone forensics technical process will aid in developing an extensive digital forensics investigation model for different types of drones. In addition, our work challenges traditional forensic analysis using open source tools because we believe that drones are always made with a number of components and different implementation of software. For instance, a drone might use different communication protocols or even storage mechanism, which requires a digital forensic examiner to be able to carve data manually to acquire evidentiary data that support the reporting phase. Particularly, this research focuses on developing a practical process for the digital evidence associated with an RPAS.

Previous research introduced a forensic analysis of the DJI Phantom 3 [6]. This paper extends these results by providing a complete technical process of drone forensics. Drone data are stored on different components of the RPAS [6], therefore, it is prudent to examine these components and develop a solid and coherent relationship between them as well as collect all digital evidence which can aid digital forensic investigators. Commercial tools (e.g., Oxygen Forensic ) are in their early stages when it comes to drone forensics. This is because of the the different types of drones which require a log parser and visualizer that supports all types of available softwares. In our preliminary work in [7], we highlighted a comparison on the installed software between three different drones and illustrated the challenges posed when it comes to development of common tools. In addition, we explored a case study using a hypothetical drone forensic tool to demonstrate the automated extraction process of GPS metadata from both media files and flight logs to generate an effective incident response procedure when dealing with illegal criminal activities using drones.

### 1.1. Contributions of the Paper

- This paper introduces an enhanced Drone Technical Forensic Investigation Process based on acquired drone data from different components of the Typhoon H.
- This paper creates a standardized approach to conduct a digital forensic analysis of a drone using the outcome of this research by proposing a digital forensic investigation into technical processes for drones.
- This paper utilizes validation and verification methods for the forensics analysis of drones. It is important to note that the validation process is critical to meet the standards of a legitimate digital forensics investigation. Therefore, several tools were tested in relation to the analysis of digital evidence found in drone components.

### 1.2. Organization of the Paper

This paper is structured as follows. In Section 2, we discuss the previous work carried out on drone forensics. Section 3 addresses the proposed technical investigation process. Next, we discuss drone forensic analysis in Section 4. Section 5 presents the GPS metadata analysis, while Section 6 presents a summary of our findings. Finally, we conclude the paper with future research directions in Section 7.

## 2. Related Work

A recent work [8] highlights some key issues confronting digital forensic practitioners, including the number of different file systems on a drone, and the seizure and analysis of different physical devices. Moreover, a proposal for the preliminary analysis of forensic challenges in RPASs is outlined in [9]. The authors also documented the analysis of the Parrot Bebob drone and discussed both essential and non-essential data found on a drone. One of the interesting conclusions of this research is that drones do not require any personal information in order to operate. Therefore, drones work differently from the normal operation of other embedded systems.

One of the most relevant works to our research is presented in [10]. The authors utilized open source tools for the forensic analysis of a multi-platform RPAS system, and they applied their analysis to two types of drones, the DJI Phantom 3 and Parrot AR Drone 2.0 as well as a mobile application platform.

In addition, Jain et al. [11] analyzed the basic structure of five commercially available drones and proposed certain steps that could aid the digital investigation of drones. The researchers made suggestions regarding the implementation of their proposed drone forensic framework. The proposed technical process includes tips such as preparation at the crime scene; identification; drone classification; checking for customization; geo-location; fingerprints; Wi-Fi; memory card; and reporting. Additionally, the phases provided by their research are extended in this paper to enhance the offline digital forensic investigation process. The authors specifically focused on the identification of physical evidence collection and the live acquisition process. It is also crucial to emphasize the importance of technical analysis and recovery of digital evidence.

Clark et al. presented the analysis of the DJI Phantom III in [12] where they developed a tool to parse and decrypt recovered flight logs. However, the interpretation of decrypted information was not discovered. The current state of the literature significantly lacks information on the interpretation of the information recovered from RPAS, such as drone flight status, accuracy of satellite signals, flight mode, error warnings, roll, and voltage. The research efforts that are discussed above concentrate on the extraction of data from different types of drones. The process presented in this paper integrates the forensic analysis of the RPAS into a technical process that can be applied to wide range of drones with similar storage mechanisms and functionality. Nevertheless, there could be different cases where drones are customized, hence require in-depth analysis and further studies.

## 3. Proposed Drone Technical Forensic Investigation Process

**Figure 1:**
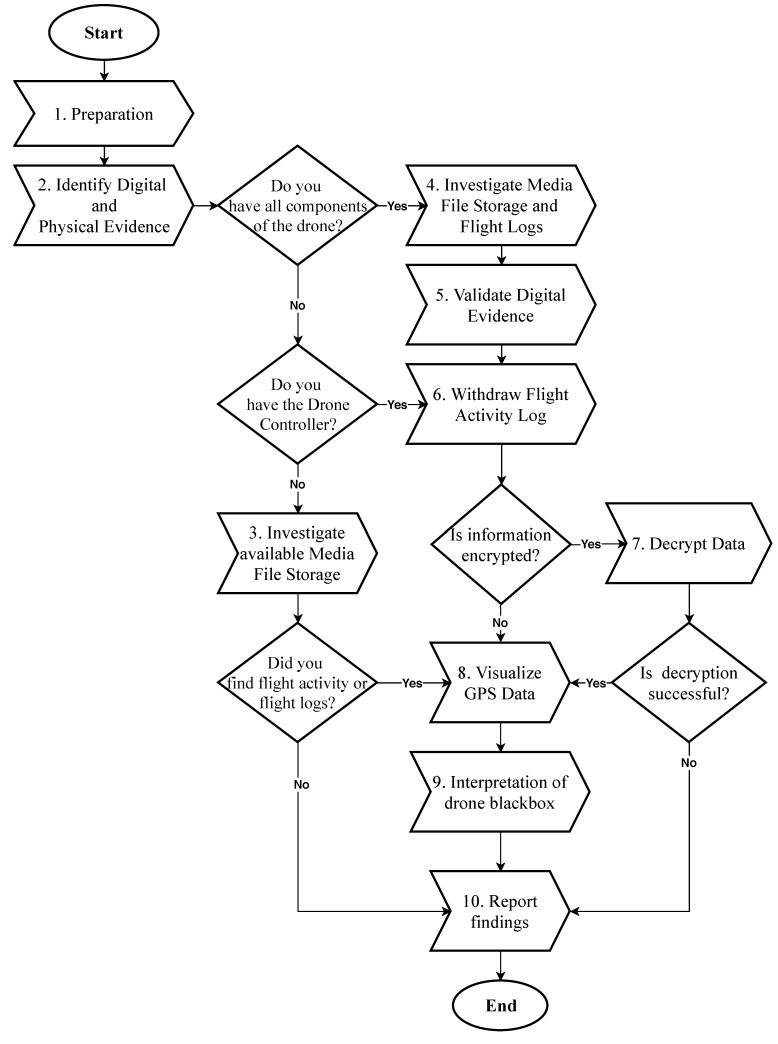
Proposed drone technical forensic investigation process.

The primary focus of this research is to generate a technical process that can be implemented by forensic examiners and law enforcement investigators. Figure 1 illustrates the ten technical phases to conduct forensic investigations on drone-related incidents. A digital forensic examiner needs to start with the “preparation” phase, which deals with the collection of evidence to the next phase as well as “identification” of both digital and physical evidence. Identifying drone components at a crime scene is a crucial step in conducting forensic investigation accordingly. Drone components can be electronic parts that are associated with drone flight operations. An example of such is the drone remote controller, which can be either built-in or in a smartphone application. The drone body contains a computer chip, cameras, and memory cards. An investigator, therefore, needs to verify the type of drone component, and, based on this information, a decision can be made. However, in some cases, the drone might contain customized hardware, which requires further determination.

Additionally, in the event that all drone components are available, a complete report of findings can be achieved. For instance, Phase 4 of the proposed technical process draws the importance of investigating media file storage and flight logs to extract relevant digital evidence and validate them accordingly, as described in Phase 5. Validation of digital evidence is crucial to make sure that data have not been altered. This can be achieved by comparing timestamps and meta-data found in media files with flight activity logs, which includes information found in media files, such as flight duration, and GPS metadata. Therefore, the analysis of both media files and flight activity logs lead to more accurate findings. However, if the collection of digital evidence is only on the controller of the drone, then investigating media file storage is not guaranteed because media files are stored in the memory cards.

Furthermore, the availability of the remote controller makes the analysis smoother. Similarly, withdrawing the flight activity log provides an extra layer to the visualization and analysis of GPS data, as shown in Phase 6; however, the storage mechanism of an RPAS stores flight logs on the remote controlled storage device. Therefore, it is difficult to recover flight activity logs from components other than the remote control. Consequently, Phase 7 demonstrates the possibility of recovering encrypted flight logs. For instance, DJI drones use encryption mechanisms to store flight logs which require decryption of these files in order to visualize GPS data. As illustrated in Phase 8, however, if decryption is not successful (especially with a customized type of drone) then that will move the process to the “reporting” phase (Phase 10). On the other hand, Phase 9 is an important phase in the proposed technical process, which has not been addressed in previous work or even non-traditional digital forensic process. This phase deals with the analysis of flight status leading to a behavioral analysis of the drone’s activity. Phase 9 is described as the “interpretation of drone black-box”, which is discussed in more details in Section 4.1.

**Figure 2:**
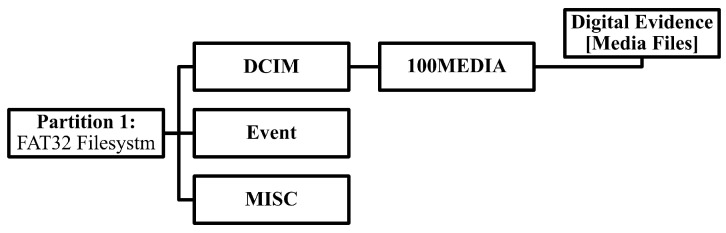
File system structure on the SD card of the Typhoon H camera.

On the other hand, if a remote controller is not available, then the investigator could apply different approaches in order to analyze media files. The file system structure of media storage is illustrated in Figure 2 where possible extraction of the media files can be achieved for further analysis. In addition, in Phase 3 of the proposed technical process, we describe that, in the condition where drone controller is unavailable, then analyzing the artifacts found in media files is necessary because they contain relevant meta-data related to the flight activity. Finally, an investigator would be able to report findings based on the proposed technical process.

## 4. Drone Forensics Investigation

This research employed the Typhoon H drone as a case study to better understand the structure of digital evidence available on drones and provides ten technical phases to support the investigation process of drone forensics. This paper divides the forensic analysis of drones into three parts, as shown in Table 1. Moving a step further, we built the findings into the drone forensic investigation technical process. Since the focus of this research is to generate an investigative technical process for drone forensic, we performed the forensic analysis using open source tools because most of the well known digital forensic tools are still in their early stages and only support certain types of drones. We conducted our analysis on the following three components of the Typhoon H drone:

- Memory Card of Camera
- Drone chip
- Controller chip

### 4.1. Tools Used in Drone Forensic Investigation

**Figure 3:**
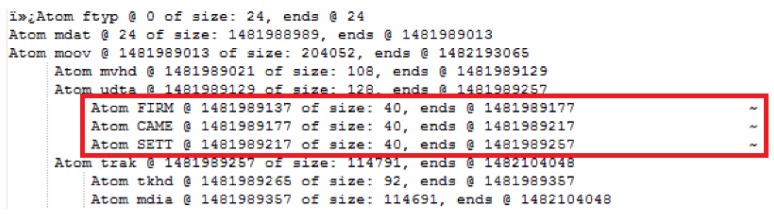
Analysis of MP4 atoms.

**Figure 4:**
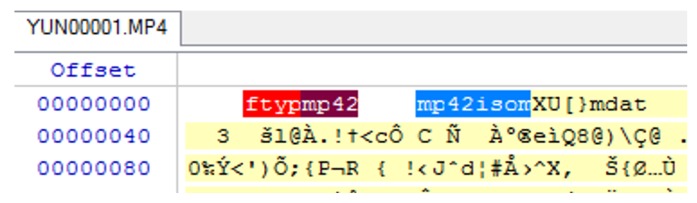
MP4 file header.

**Figure 5:**
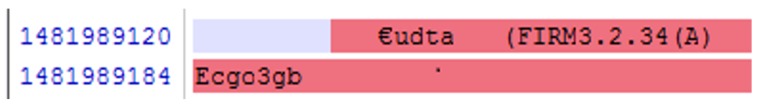
Camera information header (the vital atom/box in media files in this analysis).

**Figure 6:**
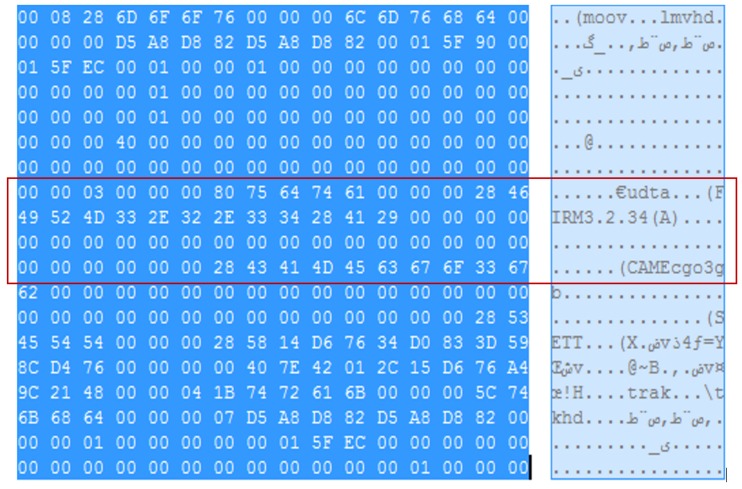
Content of “udta” box.

**Figure 7:**
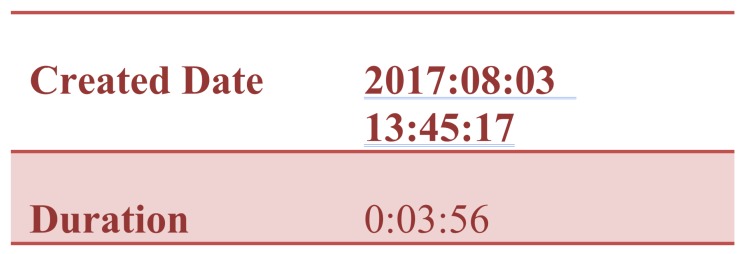
Timestamps and flight duration.

**Figure 8:**
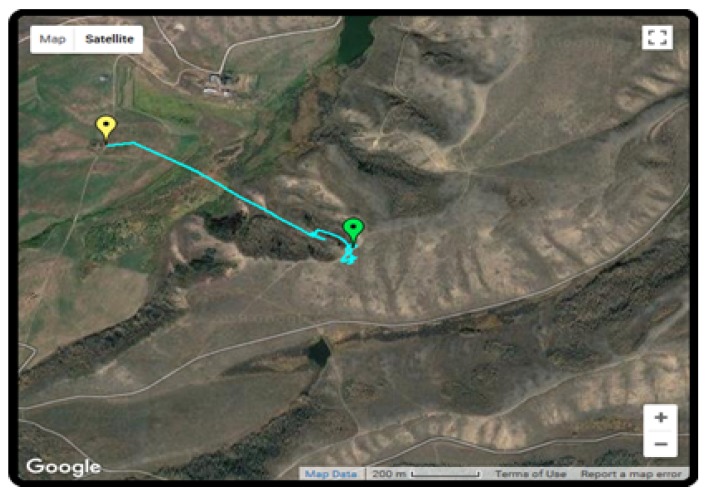
GPS metadata visualization.

**Figure 9:**
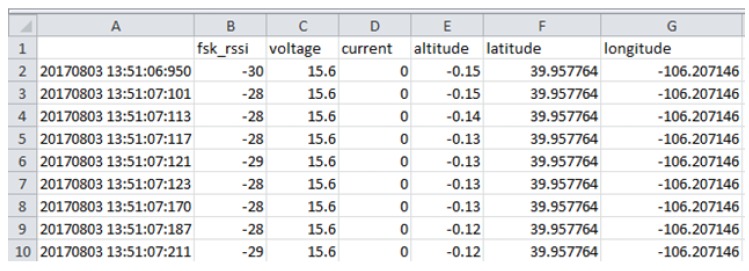
Content of flight logs.

**Figure 10:**
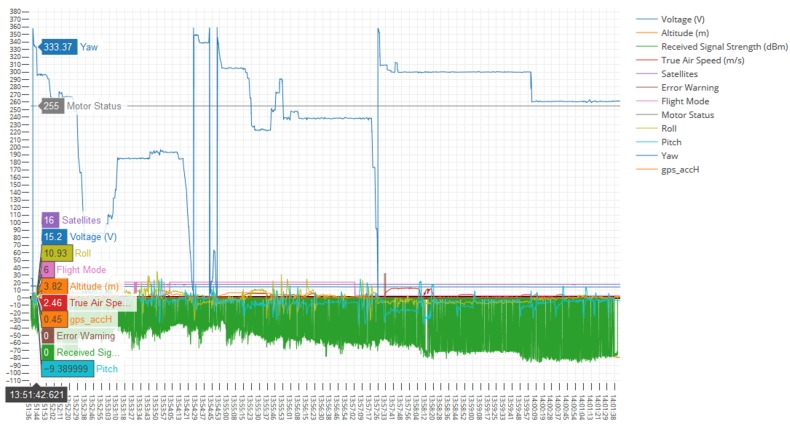
Drone black-box information.

**Figure 11:**
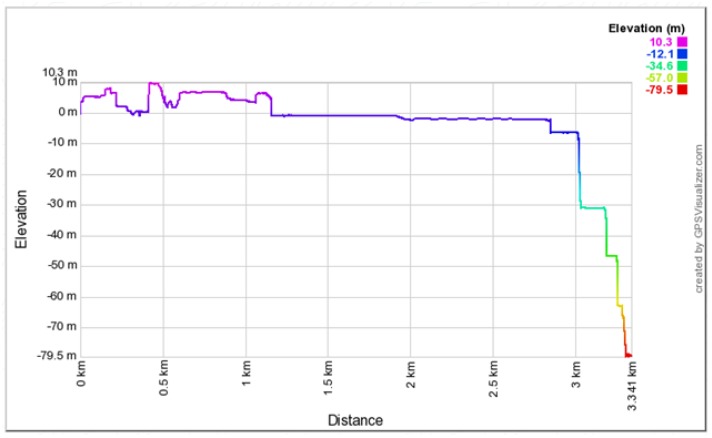
Flight activity.

**Figure 12:**
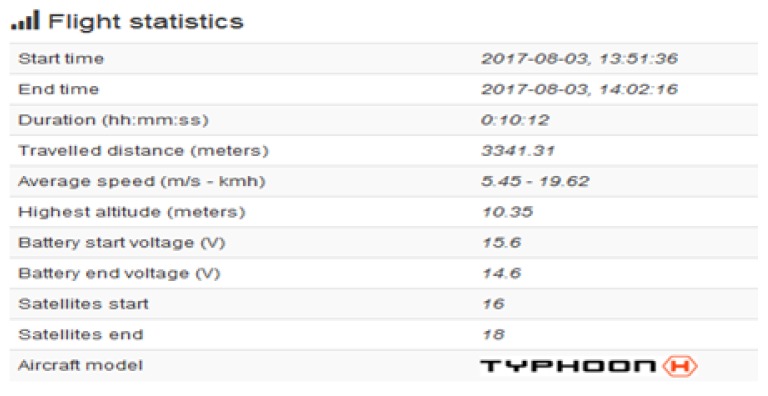
Sensor data.

The Autopsy 4.6.0 forensic tool was mainly used for data recovery, alongside some keyword searches where we analyzed the file system of each component and searched for relevant files that could aid in investigative reporting. Tools such as ExifTool 11.10, Winhex 16.7, MediaInfo 18.8.1, and 010 Editor 8.0.1 were helpful in analyzing the content of exported media files recovered from drone components. Some of the important information retrieved from these files are partially depicted in Figure 3, Figure 4, Figure 5, Figure 6 and Figure 7. Figure 3 shows three unknown atoms marked with a tilde (∼) symbol at the end of the line. These atoms are important for the verification of the camera model, firmware version, and serial number, which aids in identifying the use of any customized device attached to the drone. This draws the importance of examining media files forensically to discover anti-forensic techniques that could be used. In addition, we used Autopsy 4.6.0 to export flight logs from the controller chip of the drone to visualize GPS data, using a WebFlightPath python script, a GPS Visualizer, and Google Earth, as illustrated in Figure 8, Figure 9, Figure 10 and Figure 11. Referring back to the “interpretation of drone black-box” phase, Figure 10, Figure 11 and Figure 12 draw the interpretation of the actual flight status of the RPAS—considering the *y*-axis and *x*-axis to identify relevant information about the whole flight activity, so as to reach a better conclusion before reporting digital forensic findings. For instance, interpreting the altitude, strength of signals, and the three principal axes of an RPAS (pitch, roll, and yaw) would aid in determining if an incident was intentional (possible attack) or accidental.

### 4.2. File System Analysis and Exported Media Files

The file path location is important, because an image acquired from a MicroSD card of the camera of the drone is used in identifying that the file system is a FAT32 type, which contains all videos in the “DCIM” folder. Then, it is straightforward from that point onward. For instance, to extract all media files of type “MP4” under the following path “\1\vol_419430\DCIM\100MEDIA\YUN00001.mp4”, the first step is identifying the timestamps of any media files. Since the focus of this paper is to develop a technical process for drone forensics, the “YUN0001.MP4” media file was analyzed as a scenario. We investigated the timestamps of the media file “YUN00001.mp4” using ExifTool, written by Phil Harvey, and subsequently discussed further analysis regarding the investigation.

### 4.3. The Structure and Atoms of an MP4 File towards Manual Forensic Techniques

The MP4 file format consists of multiple atoms, and our research focuses exclusively on those atoms that are relevant to forensic investigators, especially for RPAS digital evidence. Figure 3 illustrates the atoms and the structure of the MP4 file. This would aid forensic investigators in extracting metadata by applying manual analytical techniques.

Boxes of the MP4 file are identified in a 4-byte sequence, which starts with the “ftyp” box that indicates the file type (see the file header given in Figure 4). When analyzing this type of file, the most important aspect is the metadata related to the investigation purpose, such as timestamps, camera type, and other relevant data. This can be found in the “mdat” box. The following five analysis steps provide more details about the data carving technique used on the extracted media files.

1. The first box/atom indicates the file type, which is “MP42” at offset 0–24.
2. Then, there is “mdat” atom at offset 24 of size: 1,481,988,989 bytes. The “mdat” atom usually contains media data of the video stream.
3. The “mvhd” atom starts at offset 1481989021 with a size of 108 bytes, and then the “udta” starts at offset 1481989129 with a size of 128 bytes. The “udta” atom contains two boxes, “FIRM” and “CAME”. Figure 5 and Figure 6 depict information such as firmware version and camera type. The information illustrated on Figure 6 is important to report any customization or anti-forensic techniques have been used on the drone.
4. Timestamps are very important. Extracting these timestamps would, therefore, support the forensic analysis. Timestamps are found under the “moov” atom, which contains the “mvhd” and “udta” boxes (see Figure 7).
5. We follow the timestamps carving technique below from the mp4 file. The “moov” (Movie Header) atom belongs to the top level and is an invaluable spot for digital forensic investigators. In our case, the “moov” atom starts at offset 1481989013 with a size of 20,405 bytes. The structure of the stored MP4 video contains multiple timestamps under the “moov” atom. Interestingly, during the video streaming of the drone, the timestamps are recorded in a sequence of three-second updates. The actual timestamps of the created video can be extracted using Exiftool. The command “Exiftool.exe—CreateDate—Duration YUN000001.MP4” can be used to extract metadata from the video. The above metadata extraction would help digital forensic investigators to link appropriate digital evidence in the next phase of the analysis. Our initial plan was to extract GPS metadata information from the stored videos in the SD card. However, we concluded that the Typhoon H drone employs a different storage structure and GPS metadata are not stored in the camera memory. This is an unlikely case for other types of drones such as the DJI Phantom, where media files contain GPS metadata [10]. We now move to the next forensics analysis phase, which involves looking for GPS metadata in the Typhoon H drone.

## 5. GPS Coordinates Metadata

Particularly for the studied drone, the controller has a significant amount of information that can aid in a forensics investigation. The controller of the Typhoon H drone is equipped with an Android operating system ground station and a screen attached to it. Note that most of the other known drones require the use of mobile device applications instead of an additional hardware. This feature enables the digital forensic investigator to access GPS related metadata without the need for file decryption. For instance, the GPS metadata of the DJI drone requires an investigator to decrypt the DAT file in order to collect relevant information [12]. However, in Typhoon H GPS and other examples, the flight logs are recorded in plaintext format known as CSV file. In this paper, we converted the CSV file to KMZ (Keyhole Markup Language) file format for us to be able to visualize the GPS data through Google Earth to view the flight activity, as shown in Figure 8. The conversion of the CSV file can be performed online at http://www.gpsvisualizer.com.

In Figure 9, we present the exact GPS coordinates acquired from the drone. The screenshot of the telemetry file illustrates flight activity, and it is located in the following path: “/Vol18/media/0/FlightLog/Telemetry”. Flight activities occurred on 8 March 2017 at 13:51:06:950 (−00:06 UTC) and the drone landed at 14:02:16:354 (−00:06 UTC). Figure 11 shows some further information related to specific flight activity, measuring altitude during flight activity. In addition, information such as distance and statistical flight records can be also obtained and visualized. More interestingly, the flight log file stores valuable information relevant to the status of RPAS during flight activity. Figure 10 illustrates information such as flight mode, voltage, and error warning which could be worth investigating. We used the WebFlightPath python script to parse the flight logs. This yielded a significant amount of information during the investigation of GPS metadata.

## 6. Summary of Findings

The process of drone forensics involves three main evidentiary categories. Each and every drone has propriety structures. However, the large number of file systems involved in a particular case is considered to be a technical investigation issue. This also applies to the number of acquired forensic images related to a single case. Therefore, the outcome of the analysis presented in this paper is to assist in the development of a technical process for drone forensics.

It is crucial to take the issue of anti-forensic techniques into consideration when dealing with such technology. Our preliminary results in [7] present possible drone anti-forensic and customization techniques and propose a Drone Forensic and Incident Response Plan (DFIR). Emerging technologies produce large volume of data which requires standard measures to securely handle data processing. For instance, RPASs rely on a massive number of communication commands to operate; therefore, the communication layer at this stage needs to be well compressed and encrypted to avoid possible disruption, especially with the adoption of Drone as a Service (DaaS) [13,14,15,16]. As a result, we measured the security level of the encryption mechanism of flight logs found on drones to determine the encryption and compression of transmitted data. However, the security and privacy issues of transmitted communication is out of the scope of this research as we are only concerned about the structure and layout of data that can aid digital forensic investigators. Our forensic analysis indicates that an RPAS contains a mixture of different software, file systems, and unpublished features, which creates more challenges to the investigation process; therefore, the investigator has to be familiar with the essential and non-essential digital evidence. This can enhance the identification of any anti-forensic techniques which require further exploration. Table 2 illustrates essential and non-essential digital evidence found while analyzing the drone image. More explanations regarding the content of each digital evidence is as follows:

- Media files include the extraction and analysis of videos and images.
- Flight logs include sensor metadata and GPS coordinates .
- Event logs include controller commands, connection errors, and system events.

**Figure 13:**
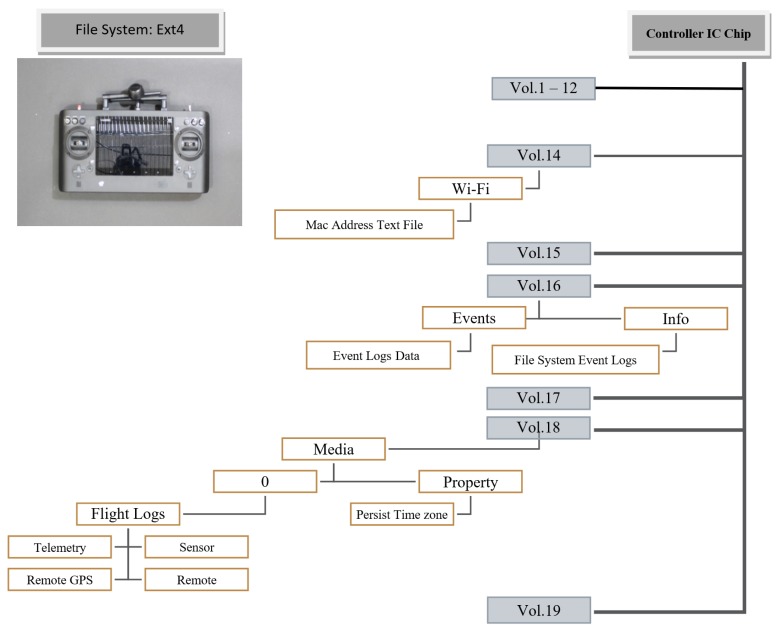
Structure of forensics evidence on Ground Station Controller.

Figure 13 shows the structure of digital evidence found in the Ground Station Controller (GSC) including sensor data, flight logs, and media files. Also, Table 2 illustrates that GSC contains a wealth of essential digital evidence related to flight activities. Analyzing the GSC would help forensic investigators to respond to complicated drone incident investigations.

## 7. Conclusions and Future Work

In this paper, we propose ten technical investigative phases for forensic analysis of RPASs, where the Yuneec, Typhoon H is used as a case study. In addition, the technical process identifies “preparation” as the first phase, which describes the preparation of digital evidence to include a collection of electronic devices at the crime scene. The next phase is the identification of both physical and digital evidence. This phase identifies the types of digital evidence including the classification of the drone. The third phase is where the forensics investigation commences, either on all components of the drone or on some of them. Based on the third phase, there will be subsequent technical steps that will need to be followed in order to achieve a complete and forensically sound report. Phases 4–10 primarily concentrate on the analysis of outcomes during the investigation, which, if adhered to, can result in a successful, and forensically sound collection of evidence and reporting.

The proposed work in this research was applied in our initial phase in generating a drone forensic tool to aid in reporting illegal flight activities. Upcoming works can be done on other types of drones based on the proposed technical process in order to develop a complete structure of drone forensics. In addition, we are currently developing open source tools which can analyze different atoms of media files as well as analyze digital evidence found on drones to enhance the validation procedure of digital evidence.

Based on the methodology discussed in acquiring forensically sound digital evidence, we deliberated more by developing a forensic tool to examine a hypothetical drone case automatically. This case deals with a specific type of incident of drones such as flying in a restricted area. Here, we assume that a drone has flown in a no-fly zone for any purpose, and law enforcement officers have seized the drone body only. The question we will answer is: How can we prove flight activities in no-fly zones in a forensically sound manner, and reach the admissibility level against criminals? Therefore, a tool that can acquire the extracted media files from the drone’s memory storage and process its GPS metadata to match it with no-fly zone location would aid in investigating the present response to such incidents. More so, in the availability of the drone control, it is important for an investigator to conduct behavioral analysis by analyzing the flight activity to interpret the “drone black-box” and reach a classification of certain measures to reflect the category of the pilot intention. On the other hand, considering drone anti-forensic and customization techniques are important aspects in drone incident response. In addition, an analysis of the secure encryption mechanism of flight logs is crucial on all commercial RPASs to enhance admissibility of drone digital evidence.

## Figures and Tables

**Table 1:** The structure of analysis tools based.

Analysis Tool	Category of Each Tool Used for
Autopsy 4.6.0	File System Analysis & Exporting Media Files
Exiftool 11.10 WinHex 16.7 MediaInfor 18.8.1 010 Editor 8.0.1	Timestamp Analysis & Structure of Media Files
WebFlightPath GPS Visualizer Google Earth	GPS Data Visualization

**Table 2:** Essential and non-essential drone digital evidence.

Drone Component	Media Files	Flight Logs	Event Logs	GPS Metadata	Sensor Logs	Flight Activity
Ground Station Controller	✔	✔	✔	✔	✔	✔
Mobile Device	✔			✔		✔
Memory Cards	✔					
Drone Chip			✔			
